# Solar Ultraviolet Radiation Risk Estimates—A Comparison of Different Action Spectra and Detector Responsivities

**DOI:** 10.3390/ijerph18094887

**Published:** 2021-05-04

**Authors:** Friedo Zölzer, Stefan Bauer

**Affiliations:** 1 Institute of Radiology, Toxicology, and Civil Protection, Faculty of Health and Social Sciences, University of South Bohemia, 37011 Ceske Budejovice, Czech Republic; 2 Federal Institute for Occupational Safety and Health (BAuA), 44149 Dortmund, Germany; bauer.stefan@baua.bund.de

**Keywords:** solar ultraviolet radiation (UVR), erythema, non-melanoma skin cancer, melanoma, action spectrum, detector responsivity, solar zenith angle (SZA), daily and annual course, correction factor

## Abstract

Studies assessing the dose–response relationship for human skin cancer induction by solar ultraviolet radiation (UVR) apply a range of methods to quantify relevant UVR doses, but information about the comparability of these datasets is scarce. We compared biologically weighted effectivities applying the most relevant UVR action spectra in order to test the ability of certain UVR detectors to mimic these biological effects at different times during the day and year. Our calculations were based on solar spectra measured at Dortmund, Germany (51.5° N) and at Townsville, Australia (19.3° S), or computed for latitudes 20° S and 50° N. Convolutions with the CIE action spectra for erythema and non-melanoma skin cancer (NMSC) and with ICNIRP’s weighting function showed comparable solar zenith angle (SZA) dependences with little influence of season or latitude. A different SZA dependence was found with Setlow’s action spectrum for melanoma induction. Calculations for a number of UVR detector responsivities gave widely discrepant absolute irradiances and doses, which were nevertheless related to those calculated with both CIE spectra by correction factors largely independent of the SZA. Commonly used detectors can thus provide quite accurate estimates of NMSC induction by solar UVR, whereas they may be inadequate to mimic melanoma induction.

## 1. Introduction

After decades of research, the dose–response relationship between solar ultraviolet radiation (UVR) and carcinogenicity in human skin is still largely unknown. We cannot predict the probability of an individual to develop some kind of skin cancer after being exposed to a certain amount of solar UVR, even if we know the spectral composition of sunlight to which he or she was exposed. There is evidence from animal experiments that UVR can induce the main types of skin cancer—squamous and basal cell carcinoma (SCC and BCC) and malignant melanoma (MM) [1,2]. Epidemiological studies show correlations between skin cancer frequencies in humans and geographical latitude [3,4,5,6], types of occupation [7,8,9], leisure behavior [10,11,12], or sun exposure patterns [13]. These are only surrogates of exposure because there is no information about important influencing factors. When geographical latitude is studied, typically with local stationary devices, we may have some measure of the amount of solar UVR reaching the Earth’s surface [14,15,16,17], but we are lacking knowledge about personal exposure times and skin orientation towards the sun. Otherwise, when personal exposure is studied, cumulative individual exposure times can be determined, sometimes even with temporal patterns, and the positioning of measuring devices at different body sites may help to account for the orientation towards the sun [18,19,20], but we do not have detailed information about the spectral composition of UVR reaching the skin. In studies of occupational or leisure exposure [21,22], and even in some studies of geographical latitude, devices providing weighted exposures are used, such as electronic photodetectors or photosensitive films, which supposedly have a spectral sensitivity close to that of human skin with respect to carcinogenicity.

Solar spectra with adequate resolution allow for computational weighting using different spectral response functions. However, spectral information is usually available only when insolation is studied at certain latitudes and times during day, year, etc., but not for the assessment of personal exposures. In order to obtain spectral details for personalized dosimetry, we would have to measure solar spectra at dozens of skin sites of hundreds of individuals, which is practically impossible. So, the question arises how well our photodetectors and photosensitive films imitate the spectral sensitivity of human skin with respect to UV induced carcinogenesis.

There is considerable evidence that the induction of non-melanoma skin cancer (NMSC) in humans has a wavelength dependence resembling that of erythema. For instance, data on skin cancer induction in mice which were corrected for differences in epidermal transmission between mice and humans resulted in an action spectrum referred to as Skin Cancer Utrecht Philadelphia, SCUP-h [23], being close to the erythema curve. This standard erythema action spectrum [24] published and repeatedly updated by the Commission International de l’Eclairage/International Commission on Illumination (CIE) is the most widely used weighting function to account for adverse health effects of UVR. More recently, CIE has also published a standard photocarcinogenesis action spectrum, which is not very different [25]. Both CIE action spectra have to be applied with care as it is the case for any standard curve. It is important to keep in mind, for instance, that they are based exclusively on research on Caucasian subjects, and that they may therefore be inapplicable for people of darker complexion. There are other sources of uncertainty, such as the necessity for standard curves to be described by simple mathematical formulae, which means they ignore evidence of any “fine structure” in the experimentally determined wavelength dependence.

In spite of their general similarity, there is no complete agreement between the NMSC action spectrum and that for erythema. Due to the steepness of both weighting functions and the solar spectrum in the wavelength region between 310 and 340 nm, the relatively subtle spectral differences of both action spectra may have a non-negligible effect on weighted exposures [23]. This is even more likely to play a role for the above-mentioned devices, which are applied to assess carcinogenicity-weighted exposures; quite substantial differences by more than an order of magnitude at critical wavelengths exist between the NMSC action spectrum and the spectral sensitivity of common UVR dosimeters [26]. There is, moreover, very little similarity between the NMSC weighting function and Setlow’s proposal for an action spectrum of melanoma induction, the latter being much shallower because it supposes a predominant influence of melanin absorption at wavelengths above 310 nm [27].

How does all of that influence risk estimates for photocarcinogenicity? If we compare weighted exposures in the course of a day and a year, or at different latitudes, we may not come to the same conclusions when we use different methods of either calculative or instrumental weighting. As mentioned, for instance, there are epidemiological studies correlating skin cancer frequencies at different latitudes with ambient UVR [3,4,5,6]. However, can these studies meaningfully contribute to establishing a universal dose-effect relationship, when they all used different weighting functions? Does a doubling of an erythemally weighted UVR dose, for instance, mean the same as twice the dose measured with a certain accumulating photodetector, and which of the two, if any, is more representative of the relative effectiveness with respect to photocarcinogenesis? This, in our view, is very important to consider when addressing the question if and to what extent epidemiological studies based on weighted UVR exposures will help to develop an algorithm predicting the risk of skin cancer for any given sunlight spectrum.

## 2. Materials and Methods

### 2.1. Solar Spectral Irradiance

Throughout the year, the spectral irradiance of the sun, $E_{\mathrm{sun}}\left(\lambda ,t\right)$, is monitored hemispherically every 5 min at Dortmund, Germany (latitude 51.498100° N, longitude 7.416576° E) by a DH-3-BI photomultiplier tube mounted at the exit port of a DTMc300 double monochromator, both from Bentham, Reading, UK. t is the correlated universal time (UTC). The solar irradiance measurement between 290 and 450 nm takes 1 min. The recording begins some time before local sunrise, $t_{i}$, and ends a few minutes after sunset, $t_{f}$; thus, it is adjusted regularly. The wavelength steps vary between 0.5 nm (290–320 nm), 5 nm (320–390 nm), 1 nm (390–392 nm), 0.075 nm (392–394 nm, used to monitor wavelength stability), 1 nm (394–400 nm), and 5 nm (400–450 nm). Both, the temperature controlled entrance optic (D6-BFS diffusor, Bentham) and the fiber optical bundle (FOP-UV, Bentham) connected to the double monochromator, are optimized for UVR transmission. Once a year, the absolute spectral irradiance of the system is calibrated with a 1000 W halogen lamp traceable to the German primary irradiance standard at the National Metrology Institute of Germany (PTB).

### 2.2. Analysis Routine

To allow for a weighting with diverse action spectra or detector responsivities, $X\left(\lambda \right)$, the solar spectral UV irradiance, $E_{\mathrm{sun}}\left(\lambda ,t\right)$, measured as described in the previous section, was interpolated yielding equidistant 1 nm spacing. The convolution

(1) $$E_{X\left(\lambda \right)}\left(t\right)=\overset{400\;\mathrm{nm}}{\underset{290\;\mathrm{nm}}{\int }}X\left(\lambda \right)\;E_{\mathrm{sun}}\left(\lambda ,t\right)\;d\lambda \;$$

was carried out for wavelengths ranging from 290 to 400 nm; thus, disregarding spectral contributions of all weighting functions below 290 nm and above 400 nm. Integrating the solar UV irradiance course for a selected day j, $E_{X\left(\lambda \right),j}\left(t\right)$, over time yields the daily UV dose, $H_{X\left(\lambda \right),j}$. Finally, the cumulative UV dose up to any day $j^{\prime }$ during the year can be estimated from

(2) $$H_{X\left(\lambda \right)}\left(j^{\prime }\right)=\overset{j^{\prime }}{\underset{j}{\int }}\overset{t_{f}}{\underset{t_{i}}{\int }}E_{X\left(\lambda \right),j}\left(t\right)\;dt\;dj.$$

For $j^{\prime }=$ 365, the annual UV dose is obtained. It must be noted that the calculations according to Equations (1) and (2) were only performed for the 1, 5, 10, 15, 20, and 25 of each month in 2018. Consequently, the annual results must be regarded as (good) approximations to the real absolute values. To allow for a better comparison with literature data, the UTC dependent results are presented as a function of solar zenith angle (SZA), which was calculated according to [28].

### 2.3. Literature Solar Spectra

Further calculations were carried out on the basis of spectra published by Bernhard et al., 1997 [29]. They were measured on January 11, 1996 in Townsville, Australia (latitude 19.33° S) at different SZA. The spectrum for 20° SZA begins at 290 nm whereas the spectra for 30°, 40°, 50°, 60°, and 70° SZA at somewhat longer wavelengths up to 297.5 nm. The smallest measured spectral irradiances at these marginal wavelengths were 0.01–0.02 mW/(m^2^ nm). The table provided in their paper contains values at 0.5 nm steps up to 300 nm, 1 nm steps up to 310 nm, 2 nm steps up to 340 nm, 5 nm steps up to 400 nm, and 10 nm steps up to 650 nm. We calculated effective irradiances in steps of 1 nm; therefore, we had to interpolate their spectra.

Finally, computed solar spectra were taken from the “UV-B Handbook” by Gerstl et al., 1983 [30]. It contains spectral irradiances for different latitudes, SZAs, and ozone concentrations—but only at 7 wavelengths between 290 and 320 nm. These spectra had to be interpolated at 1 nm steps as well, and extended above 320 nm. Regarding this extension, it turned out that the values of spectral irradiance given by Gerstl et al. [30] for 20° southern latitude and different SZA matched those of Bernhard et al. [29]. Consequently, it was assumed that for other latitudes as well, the spectra of Bernhard et al. could be scaled to the values of the Gerstl spectra at 320 nm and used as continuation of computed spectra above that wavelength. Above 320 nm, the form of the sun’s spectrum does not depend very strongly on the SZA [29], in contrast to the overall global irradiance. In addition, the effective wavelengths for carcinogenicity are mainly below 320 nm. Thus, possible errors due to our particular choice of extension should be of minor importance.

### 2.4. Action Spectra

As indicated in the introduction, several biological effects like erythema and NMSC induction as well as physical or chemical effects supposedly imitating the wavelength dependence of the biological effects are compared in the following.

**Figure 1:**
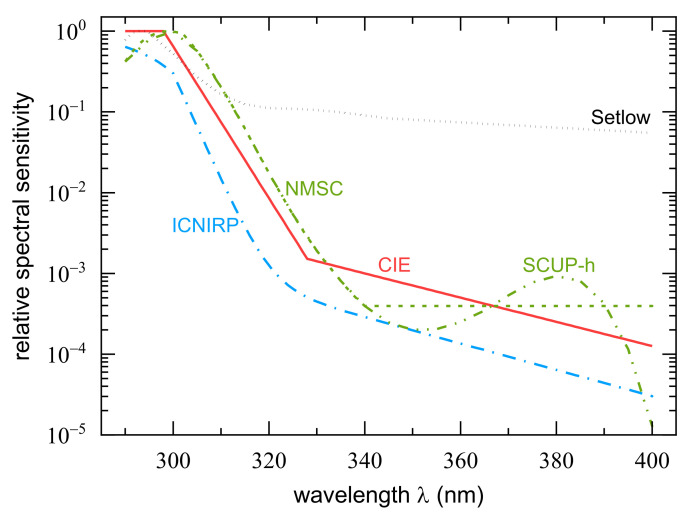
Semilogarithmic presentation of the CIE standard curves for erythema [24] and for non-melanoma skin cancer (NMSC) [25], the latter with one of its predecessors, the Skin Cancer Utrecht-Philadelphia action spectrum modified for human epidermal absorption (SCUP-h) [23]. ICNIRP’s UVR hazards reference weighting function [31] and the cutaneous melanoma sensitivity proposed by Setlow [27] are also presented. Further information is given in Table 1 below.

The action spectrum for carcinogenicity in humans, i.e., for the induction of non-melanoma skin cancer cannot be determined experimentally for ethical reasons. One important data set whose estimates are based on is the Skin Cancer Utrecht-Philadelphia (SCUP) action spectrum, which reflects spectral variation of skin cancer induction in hairless mice [23]. It is referred to as SCUP-m, and used to calculate a slightly modified version for humans called SCUP-h taking differences in epidermal transmission between hairless mice and humans into account, see Figure 1. The resulting spectrum has a maximum at 300 nm, declines more or less exponentially towards a minimum at 350 nm with 0.002 relative sensitivity, swings back by a factor of almost 5 to a maximum at 380 nm, and decreases rapidly towards longer wavelengths. The original publication [23] contains numerical values at 5 nm intervals between 254 and 400 nm, which were interpolated semi-logarithmically in 1 nm steps for this work. The CIE standard curve for non-melanoma skin cancer induction (NMSC) [25], see Figure 1, is identical with SCUP-h below 340 nm, but instead of the switchback above that wavelength it assumes a flat response up to 400 nm. As the NMSC and SCUP-h weighting functions are so close to each other, all weighted solar irradiances in the daily and in the annual course of 2018 measured at Dortmund differ by less than 4%; thus, only the standardized NMSC action spectrum will be applied for the analysis presented hereafter.

The most widely used action spectrum for erythema induction in humans is the standardized action spectrum published by CIE [24]. A parametrization of this spectrum is given by

(3) $$X_{\mathrm{CIE}}\left(\lambda \right)=\left\{\begin{array}{c} 1.0\;\;\;\;\;\;\;\;\;\;\;\;\;\;250\;\mathrm{nm}\leq \lambda \leq 298\;\mathrm{nm}\\ 10^{0.094\left(298-\lambda \right)}\;\;\;\;\;298\;\mathrm{nm}<\lambda \leq 328\;\mathrm{nm}\\ 10^{0.015\left(140-\lambda \right)}\;\;\;\;\;328\;\mathrm{nm}<\lambda \leq 400\;\mathrm{nm} \end{array}\right.$$

i.e., three parts can be distinguished: a constant region from 250 to 298 nm, an exponential decline between 299 and 328 nm, which is essentially parallel to the NMSC curve but shifted about 5 nm to shorter wavelengths, and a less steep though still exponential decline between 329 and 400 nm. The CIE erythema action spectrum is based on a proposal by McKinlay and Diffey, 1987 [32], which combined experimental data available at that time and statistical considerations to produce an “average erythema curve”. There are slight differences between that proposal and the final CIE standard curve, but they cause differences in weighted irradiances of no more than 2–3% [33]. A review of the historical erythema action spectrum development can be found, for example, in a paper by Schmalwieser et al. [34]. In addition, the CIE erythema action spectrum is the one used for calculations of the UV index [35].

The International Commission on Non-Ionizing Radiation Protection (ICNIRP) regularly addresses occupational hazards originating from exposures to optical radiation. In order to simplify workplace protection against detrimental effects of UVR, ICNIRP reports an action spectrum [31] that accounts for adverse UVR health effects to the human skin and to the eyes. Consequently, the superposition of several action spectra and subsequent normalization lead to an overall shift to shorter wavelengths accompanied by relatively less sensitivity compared to CIE’s standard erythema curve, but the slopes of both exponential declines for λ> 300 nm are very much alike. The maximum of ICNIRP’s relative spectral sensitivity for UVR hazards lies at 270 nm and does not contribute to the weighting of solar UVR. Similar to $X_{\mathrm{CIE}}$, a mathematical description exists for the ICNIRP weighting function that is given by [31]

(4) $$X_{\mathrm{ICNIRP}}\left(\lambda \right)=\left\{\begin{array}{c} 1-0.36\times {\left(\frac{\lambda -270\;\mathrm{nm}}{20\;\mathrm{nm}}\right)}^{1.64}\;\;\;\;\;\;\;\;\;\;\;\;270\;\mathrm{nm}<\lambda \leq 300\;\mathrm{nm}\\ 0.3\times 0.736^{\left(\lambda -300\;\mathrm{nm}\right)}+10^{\left(2-0.0163\times \lambda \right)}\;\;\;\;\;300\;\mathrm{nm}<\lambda \leq 400\;\mathrm{nm} \end{array}\right.$$

For melanoma induction, a very different action spectrum was proposed by Setlow et al., 1993 [36]. The authors investigated melanoma induction in fish of the genus *Xiphophorus*, and found a much flatter action spectrum than was (and is still) assumed for NMSC induction. They also noted the similarity of their action spectrum with melanin absorption above 310 nm, and suggested that a much greater part of melanoma might be induced by UV-A and visible radiation of the solar spectrum than is the case for NMSC. In 1999, Setlow complemented the older set of data with results from additional experiments at 547 nm, and again pointed to the possible role of melanin absorption for melanoma induction [27]. Since his fish data have very large uncertainties, we used the “hypothetical action spectrum” from the latter publication. It is based on the combined absorption spectrum of DNA and melanin and corrected for human epidermal absorption according to [37]. In order to assess how critical might be the particular assumptions about epidermal absorption, we carried out some calculations with epidermal absorption data from [38]—the same data as were used for the correction of the SCUP-m action spectrum (see above)—and data from [39] for unexposed skin. The resulting spectrum, i.e., the combined absorption spectrum of DNA and melanin corrected with these alternative epidermal absorption data, is very similar to what is presented in Figure 1; thus, it is not shown.

### 2.5. Detector Responsivities

**Figure 2:**
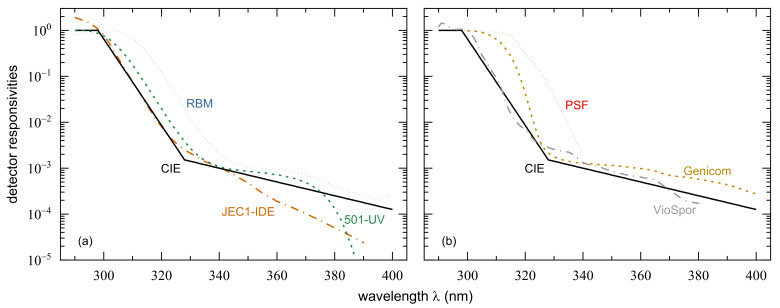
Normalized detector responsivities of (**a**) Robertson–Berger meters (RBM) [41], Solar Light’s Model 501-UV [42], and of the silicon carbide photodiode (JEC1-IDE) used by Heydenreich et al. [43]. (**b**) Relative spectral response of the Genicom UVB dosimeter employed by Grandahl et al. [44], of a 40 µm thick polysulphone film (PSF) [45], and VioSpor’s mutant strain UVR detection system [46]. The CIE erythema reference action spectrum [24] is depicted in both panels for better comparison. Further information is given in Table 1 below.

The first widely used device having a similar spectral sensitivity as NMSC induction was the Robertson–Berger meter [40]. Evidence has been presented that not all RBMs show the same wavelength dependence, and that at least part of the variation is due to aging of the instruments [41]. However, the variation seems to appear predominantly in the spectral region above 335 nm; therefore, it is not really critical for our purposes here. A typical RBM spectrum has a maximum at 300 nm and falls off more or less exponentially between 315 and 335 nm, see Figure 2a. Its slope is similar to that of CIE’s erythema curve, but it is shifted to longer wavelengths by about 14 nm. In general, it also resembles the standard erythema weighting function for wavelengths longer than 335 nm but with higher relative sensitivity.

More recently, devices have been developed with spectral responsivities much closer to the CIE standard curve. The UV Biometer 501, abbreviated by 501-UV hereafter, is a modified version of the RBM with higher temperature stability and improved filtering [42,47]. Its sensitivity has a maximum at 292 nm, see Figure 2a, and the decline towards longer wavelengths is only shifted by about 5 nm against the CIE standard curve, in contrast to the 14 nm for RBM. Above approximately 335 nm, the spectral responsivity of 501-UV is close to the CIE standard erythema weighting function followed by a strong decrease for λ> 370 nm.

Heydenreich and Wulf, 2019 [43] achieved a better match of the CIE erythema action spectrum with their JEC1-IDE sensor, which is based on a silicon carbide photodiode. Its spectral responsivity, see Figure 2a, deviates from the CIE standard curve by just a few percent between 298 and 325 nm. It has a somewhat higher sensitivity at uncritical wavelengths below 297 nm and a steeper slope beyond 325 nm, underestimating erythema induction at longer wavelengths.

A different approach was taken by Grandahl et al., 2017 and 2018 [44,48]. They did not try to match the erythema curve as closely as possible but were mainly interested in a device, which would distinguish between UV-B and UV-A radiation. Their aluminum gallium nitride detector, denoted as Genicom, see Figure 2b, has a constant sensitivity between 280 and 305 nm, a broader but steeper “shoulder” than the CIE curve between 305 and 325 nm, and a slightly flatter response at longer wavelengths. The spectrum contained in their earlier publication was even steeper with a cut-off at about 307 nm. Later, this was recognized to be based on incorrect information from the manufacturer (K. Grandahl, personal communication).

The only action spectrum for photochemical reactions taken into consideration for this work is the one for the absorption change at 330 nm of polysulphone films (PSF), see Figure 2b. This sensitivity curve was originally suggested for a weighted measurement of UVR exposure by Davis et al., 1976 [49]. There are slight differences between his and the spectra of some other authors [50,51]. CIE has published a standard curve for polysulphone sensitivity [45]. The spectral response of a PSF film is highest between 290 and 300 nm, decreases to about half maximum between 317 and 318 nm beyond which it falls off roughly parallel to the erythema curve but with a shift of about 17 nm towards longer wavelengths. No data λ > 340 nm are given in [45].

Finally, we included in our calculations an action spectrum for the inactivation of bacterial spores (*Bacillus subtilis*). A mutant strain spore film combined with a cut-off filter (50% transmission at 320 nm) was suggested as an inexpensive personal UVR dosimeter, denoted as VioSpor. Its spectral response function [46] resembles other biological action spectra, see Figure 2b, but is slightly steeper than the CIE standard erythema curve between 300 and 320 nm. Its slope is similar to that of the CIE weighting function at longer wavelengths. For λ< 290 nm, the relative spectral sensitivity is smaller than indicated by the standard CIE erythema curve; however, these wavelengths are not taken into account for a convolution with solar spectra.

Table 1 summarizes all action spectra and detector responsivities used in this work. Additional information is listed, such as the main procedure of data processing. Whenever necessary, discrete sensitivities were interpolated in 1 nm steps on a logarithmic ordinate. It is important to emphasize that a linear interpolation would yield slightly different weighted UV irradiances. We did not check for such effects systematically, but decided to apply semi-logarithmic data interpolation consistently.

The action spectra and detector sensitivities were used in their published form, i.e., normalized to their respective maxima, which were found to be at different wavelengths, see Table 2. We respected this normalization so that comparisons with published data would be straightforward. In contrast, if all action spectra had been normalized to their sensitivities obtained at 297 nm, the wavelength considered to be the most effective for erythema induction, the weighted UV irradiances would differ with regard to the peak normalized ones by certain factors, which are given in Table 2. These renormalization factors can be determined from the original action spectrum’s sensitivity at 297 nm. The resulting weighted irradiances and doses would then be equally effective to the same irradiances and doses applied in the form of monochromatic radiation of 297 nm—provided, of course, that the action spectrum correctly describes the wavelength dependence of the effect under investigation.

Last but not least, we would like to emphasize that what we present here is the result of calculations based on published detector responsivities. The radiometers used in the studies we refer to may, for example, have differently cosine-corrected entrance optics. Moreover, some of the detecting systems employed for personal UVR dosimetry may have additional “inaccuracies” caused by individual exposure conditions including orientation and shading of the entrance optic.

## 3. Results

### 3.1. Weighted UV Irradiances as a Function of the Solar Zenith Angle

**Figure 3:**
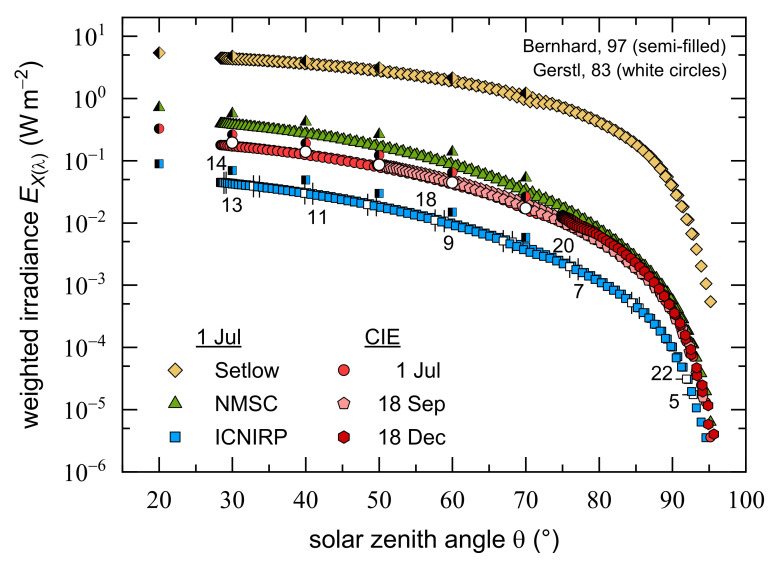
Semi-logarithmic presentation of SZA dependent UV irradiances measured on the 1 July 2018 (cloudless day) at Dortmund, Germany (latitude 51.5° N), and weighted either with CIE’s reference erythema action spectrum [24], the NMSC weighting function [25], ICNIRP’s relative spectral sensitivity for UVR hazards [31], or the melanoma action spectrum proposed by Setlow [27]. Erythemally weighted irradiances for cloudless days close to 2018′s autumn equinox (23 September) and winter solstice (21 December) are compared to those from 1 July 2018. White squares highlighted by either vertical or horizontal dashes and labelled with numbers mark full daytime hours (local time). In addition, weighted irradiances based on data from Bernhard et al. [29] (semi-filled symbols, latitude 19.3° S, 11 January 1996) and Gerstl et al. [30] (white circles, latitude 50° N) are also depicted.

Although the 2018 summer solstice at Dortmund, Germany was on 21 June, examining several days around that date revealed that 1 July was the closest one to being cloudless. Thus, it was chosen to analyze the effect of different action spectra on the daily course of weighted UV irradiances as a function of SZA, $E_{X\left(\lambda \right)}\left(\Theta \right)$, see Figure 3. Due to its comparatively flat spectral distribution in the UVA and UVB region in comparison with other weighting functions, see Figure 1, $E_{\mathrm{Setlow}}\left(\Theta \right)$ is more than one order of magnitude higher than $E_{\mathrm{NMSC}}\left(\Theta \right)$, $E_{\mathrm{CIE}}\left(\Theta \right)$, and $E_{\mathrm{ICNIRP}}\left(\Theta \right)$, but the latter three also vary significantly from each other reflecting differences in the wavelength dependences of their relative spectral sensitivities. The maxima, all located at that day’s minimum SZA of 28.4° (13:36), are given by 4.450 Wm^−2^ (Setlow), 0.393 Wm^−2^ (NMSC), 0.178 Wm^−2^ (CIE), and 0.045 Wm^−2^ (ICNIRP). The $E_{X\left(\lambda \right)}\left(\Theta \right)$ curves resemble each other but $E_{\mathrm{Setlow}}\left(\Theta \right)$ was flatter for most of the day, with the exception of the early morning and late afternoon. For example, while $E_{\mathrm{Setlow}}=$ 4% of its daily maximum for Θ= 85° (6:00), $E_{\mathrm{CIE}}$, $E_{\mathrm{NMSC}}$, and $E_{\mathrm{ICNIRP}}$ were below 1%. Note that Figure 3 depicts weighted irradiances some time before sunrise and after sunset. The morning and afternoon data strongly overlapped for each $E_{X\left(\lambda \right)}\left(\Theta \right)$ curve due to the high SZA symmetry.

In addition to 1 July 2018, two additional days are included in Figure 3; however, solely focusing on erythemally weighted UV irradiances so as not to clutter the figure. The 18 September and 18 December were the closest cloudless days in 2018 to the autumn equinox (23 September) and the winter solstice (21 December), respectively. $E_{\mathrm{CIE}}\left(\Theta \right)$ was also calculated for 25 March. The results were very similar to those for 18 September and are therefore not depicted, again in the interest of visual clarity. The general shapes of the SZA dependent $E_{\mathrm{CIE}}\left(\Theta \right)$ remain much the same throughout the year, but the weighted UV irradiances for the autumn equinox and for the winter solstice vary on a much smaller SZA scale than it is the case for the summer solstice. The maximum values differ approximately by an order of magnitude: 0.178 Wm^−2^ (1 July), 0.087 Wm^−2^ (18 September), and 0.013 Wm^−2^ (18 December). The SZA and temporal locations of these maxima shift from 1 July (Θ= 28.4° at 13:36) to 18 Sept (Θ= 49.7° at 13:18) and to 18 December (Θ= 75.0° at 12:06). Note the shift in the local maximum time for the 18 December that is caused by the biannual time change from UTC + 2 h to UTC + 1 h and vice versa at Dortmund, Germany (change to daylight saving time between end of March and end of October). For 25 March (data not shown), the maximum of 0.066 Wm^−2^ is found at Θ= 49.7° (13:18); thus, matching the autumn equinox SZA.

To gain insights into the importance of latitude in this context, solar spectra from Bernhard et al. [29] measured in Townsville, Australia (latitude 19.3° S) on 11 January 1996, and calculated ones for different latitudes from Gerstl et al. [30] were biologically weighted as well. Data were available for only a few SZAs, but as is apparent from Figure 3, the weighted irradiances are comparable to those obtained with the solar spectra measured at Dortmund, Germany. For example, $E_{\mathrm{CIE}}\left(\Theta \right)$ for latitude 51.5° N agrees within 1–12% with the weighted irradiances based on Gerstl’s calculated spectra [30] for latitude 50° N, but are about one third smaller than those determined with Bernard’s data for latitude 19.3° S. The latter match $E_{\mathrm{CIE}}\left(\Theta \right)$ based on Gerstl’s calculated spectra for latitude 20° S within a range of (4 ± 6)% (data not shown). The difference between latitude 20° S and 50° N is probably due to the slightly lower stratospheric ozone concentration at lower latitudes [29]. The agreement of weighted irradiances based on solar spectra of different origins is quite reassuring. It also demonstrates that the low-resolution spectra of Gerstl et al. [30] are good enough for dose estimates under clear sky conditions.

The CIE, NMSC, and ICNIRP curves are essentially parallel, while $E_{\mathrm{Setlow}}\left(\Theta \right)$ is decidedly flatter for most of the SZA range. In their daily courses between SZAs of 20° and 70°, CIE, NMSC, and ICNIRP weighted irradiances vary by a factor of 12–15, whereas $E_{\mathrm{Setlow}}\left(\Theta \right)\;$ changes only by a factor of about 4. This is due to the flatness of the Setlow action spectrum in the UVA region, which gives much more weight to wavelengths above 310 nm region compared to the other action spectra. As mentioned in Section 2.2, our weighting procedure disregards spectral contributions above 400 nm. For the CIE, NMSC, and ICNIRP action spectra, this is sufficient, because no spectral sensitivities are defined at longer wavelengths. Additional calculations with the solar spectra by Bernhard et al. [29] showed, however, that in the case of Setlow’s action spectrum the weighted irradiances including spectral contributions up to 550 nm are by a factor of 2.6–3.0 higher than those given in Figure 3. This factor is essentially independent of the SZA.

In summary, similar SZA dependences were found for CIE, NMSC, and ICNIRP weighted irradiances with absolute values varying by an order of magnitude, whereas irradiances weighted with Setlow’s action spectrum for melanoma increased less steeply with decreasing SZA for most of the day. Calculations based on literature data showed consistent SZA dependences virtually irrespective of season and latitude when compared with those based on solar spectra measured at Dortmund, demonstrating the usefulness even of low-resolution data.

### 3.2. Annual Course of Cumulative Weighted UV Doses

Annual biologically relevant UV doses were calculated according to Equation (2) with the CIE, NMSC, ICNIRP, and Setlow weighting functions. It is important to note that not all 365 days of 2018 were analyzed but only the 1st, 5th, 10th, 15th, 20th, and 25th of each month and some additional days, for example, being close to summer and winter solstice. Furthermore, due to this selection of roughly every 5th day, no longer just cloudless days could be used for the analysis, and the cumulative UV dose, $H_{X\left(\lambda \right)}\left(j^{\prime }\right)$, must be regarded as an approximation, albeit a sufficiently appropriate one.

**Figure 4:**
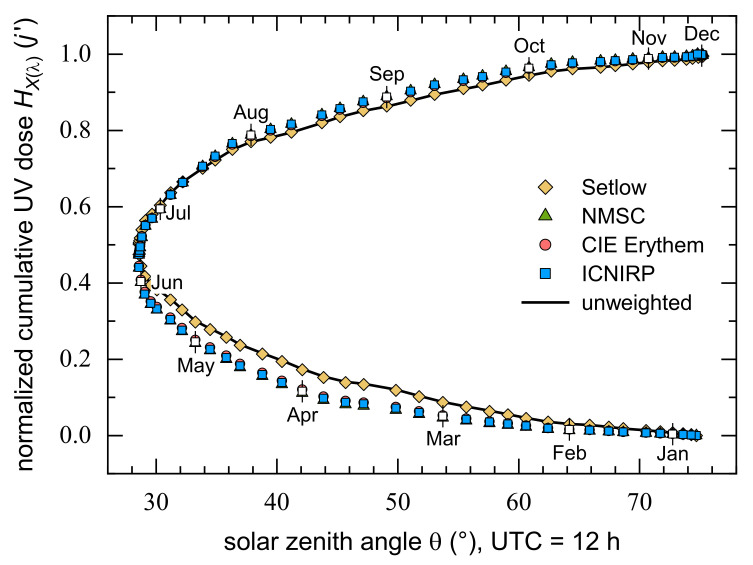
Normalized (31 December) cumulative UV doses, $H_{X\left(\lambda \right)}\left(j^{\prime }\right)$, calculated for 2018 according to Equation (2) with the Setlow, NMSC, CIE, and ICNIRP action spectra presented in Figure 1, and shown as a function of SZA, determined for UTC = 12 h, in the course of the year. The solid line represents the normalized radiometric (unweighted) annual UV dose. White symbols highlighted by vertical dashes mark the 15th of each month.

The similarity found for the daily courses (1 July 2018) of SZA dependent UV irradiances, $E_{X\left(\lambda \right)}\left(\Theta \right)$, weighted either by the CIE, NMSC, or ICNIRP action spectrum, see Figure 3, reappears for their cumulative annual UV doses, see Figure 4, which are peak normalized (31 December) for better comparison. These three $H_{X\left(\lambda \right)}$ curves are hardly distinguishable because of their strong overlap. In contrast, Setlow’s melanoma action spectrum attributes a higher relative carcinogenicity to solar UVR in the first months of the year (February to June) and a lower one between August and November. Due to the comparably flat spectral distribution of Setlow’s weighting function above 310 nm, $H_{\mathrm{Setlow}}\left(j^{\prime }\right)$ closely matches the radiometric (unweighted) annual UV doses with a percentage deviation of −2.5–0.1% throughout the whole year. By the end of March, both the radiometric and the Setlow-weighted solar spectra reach about 13% of their annual UV doses, whereas for all other weighting functions only about 8% is accumulated. This is due to the fact that the SZA dependences of the radiometric and Setlow-weighted irradiances are generally flatter than those with the CIE erythema and NMSC or ICNIRP action spectra, see Figure 3. Consequently, the UVR doses accumulated at higher SZAs during the first quarter of the year are more significant for the radiometric and Setlow-weighted annual doses than is the case with the other three weighting functions.

Half of the CIE, NMSC, and ICNIRP weighted annual UV doses were accumulated until 28 June (day 179, Θ= 28.7°) or until 25 June (day 176, Θ= 28.6°) for $H_{\mathrm{Setlow}}\left(j^{\prime }\right)$, both days being close to the middle of the year (182.5 days). There exist rough symmetries regarding 25% and 75% of the total cumulative weighted UV doses, which were found at −44 and +40 days (NMSC, CIE, ICNIRP: day 135, Θ= 33.3° and day 219, Θ= 35.4°) or at −52 and +46 days (Setlow: day 124, Θ= 36.1° and day 222, Θ= 36.3°). Without further analysis it remained unclear if the discrepancies between 25% and 75%, 4 and 6 days, respectively, result from the limited number of days for which calculations were carried out or from specific conditions during 2018 (cloudiness, ozone levels, etc.). The absolute annual UV doses amount to 18.1 MJm^−2^ (Setlow), 1.0 MJm^−2^ (NMSC), 484 kJm^−2^ (CIE), and 113 kJm^−2^ (ICNIRP). The differences between these values are substantial with, for example, a nine times higher annual $H_{\mathrm{NMSC}}$ compared to $H_{\mathrm{ICNIRP}}$.

The main notion here is that throughout the year, the cumulative UVR doses weighted with the CIE, NMSC, or ICNIRP action spectra are practically identical (when normalized to their value at the end of the year). Therefore, if necessary, they can be converted into each other by means of constant factors. In contrast, the annual SZA course of normalized Setlow weighted doses agrees with that for radiometric values.

### 3.3. UV Doses Measured by Detectors Mimicking Erythema Sensitivity

In the last decades, quite a number of measurement devices have been developed aiming to mimic the skin’s UVR sensitivity, more particularly the wavelength dependence of erythema induction. A selection of detector responsivities is presented in Figure 2 with additional information listed in Table 1. In order to assess the accuracy of these detectors to reflect erythema response, but also for an easier comparison between them, the standardized CIE erythema action spectrum and the associated weighted UV irradiances and doses were used for reference. Instead of more “sophisticated” ways of analysis, we decided to focus on the simple ratio of $E_{X\left(\lambda \right)}$ to $E_{\mathrm{CIE}}$ or $H_{X\left(\lambda \right)}$ to $H_{\mathrm{CIE}}$, hereafter referred to as $r_{X\left(\lambda \right),\mathrm{CIE}}$, because of its higher practical benefit.

**Figure 5:**
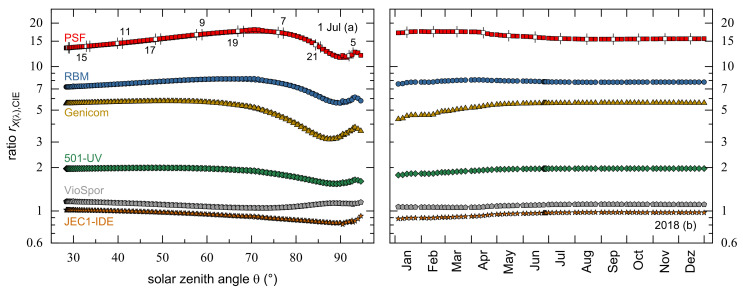
(**a**) Semi-logarithmic presentation of the ratios of measured irradiances, $E_{X\left(\lambda \right)}$, from several detecting systems with regard to those weighted with the CIE standard erythema curve, $E_{\mathrm{CIE}}$, for 1 July 2018. (**b**) Ratios of the daily UV doses for the same detectors as presented in panel (**a**) but depicted as a function of days $j^{\prime }$ in the course of the year 2018. White symbols highlighted with vertical dashes mark (**a**) local daytime and (**b**) the 15th of each month.

Figure 5a shows this ratio in the course of 1 July 2018 for six detecting systems. All depicted ratios have more or less pronounced minima in the early morning (before 6:00) and late afternoon (after 21:00), i.e., Θ> 85°, except for $r_{\mathrm{VioSpor},\mathrm{CIE}},$ which is smallest for a SZA of approximately 70°. The associated measured irradiances, however, do not contribute significantly to the daily UV doses because more than 90% of them are accumulated for Θ≤ 60°. None of the detectors shows a substantial difference between morning and afternoon, i.e., both $r_{X\left(\lambda \right),\mathrm{CIE}}$ curves overlap strongly. The SZA dependences of the ratios are rather small with mean values and standard deviations of 15 ± 2 (PSF), 7.4 ± 0.8 (RBM), 5.0 ± 0.9 (Genicom), 1.9 ± 0.2 (501-UV), 1.11 ± 0.04 (VioSpor), and 0.94 ± 0.07 (JEC1-IDE). Very similar values were found when calculations were based on solar spectra from Bernhard et al. [29], namely 13 ± 2 (PSF), 7.3 ± 0.8 (RBM), 5.5 ± 0.3 (Genicom), 2.0 ± 0.1 (501-UV), 1.15 ± 0.06 (VioSpor), and 1.00 ± 0.04 (JEC1-IDE). This demonstrates the need for a careful handling of data from weighting UVR detectors. For example, PSF detectors, which have been the most frequently applied accumulating UVR dosimeters for decades, overestimate erythemally weighted UVR exposures by a factor of about 15; thus, these values must be corrected downwards. It is also obvious that PSF has a somewhat different dependence on SZA than the CIE erythema sensitivity curve, so that the precise correction factor is 17 at 60° but 14 at 30°. For RBM, this variation is between 8 at 60° but 7 at 30°. Other devices, for example, the VioSpor or the JEC1-IDE system, are able to mimic the convolution of UVR with the CIE action spectrum quite well as both of their ratios are close to 1.

In general, ratios similar to $r_{X\left(\lambda \right),\mathrm{CIE}}$ can be calculated for the same detectors but with other action spectra than the CIE erythema standard curve as reference. However, the weighting function for melanoma induction proposed by Setlow [27] is the only one that has a markedly different spectral sensitivity; thus, one would expect an altered daily course to that presented in Figure 5a only for $r_{X\left(\lambda \right),\mathrm{Setlow}}$. Indeed, these ratios are not more or less independent of the position of the sun as it was found for $r_{X\left(\lambda \right),\mathrm{CIE}}$ but decrease with increasing SZA. For 30° ≤Θ≤ 70° of 1 July, $r_{\mathrm{PSF},\mathrm{Setlow}}$ and $r_{\mathrm{RBM},\mathrm{Setlow}}$ are roughly halved (factors of 1.8 and 2.1), whereas the ratios of the Genicom (2.6), the 501-UV (2.5), the JEC1-IDE (2.7), and the VioSpor detectors (2.7) are reduced somewhat more. These values were calculated with the spectral UV irradiances measured at Dortmund, but again, very similar values were found with the solar spectra published by Bernhard et al. [29], namely 1.7 and 2.0 for PSF and RBM, 2.4 for Genicom, 2.5 for 501-UV, 2.7 for JEC1-IDE, and 2.9 for VioSpor.

In addition to UVR detectors, which are applied for a certain period like a few hours, days or weeks to record an individual’s personal exposure, other devices are permanently installed, for instance on roofs, to continuously monitor the solar spectrum in a particular locality and to calculate erythemally weighted UVR exposure. Therefore, it makes sense to take a closer look at the variation of the dose ratios, i.e., $H_{X\left(\lambda \right)}\left(j^{\prime }\right)$ to $H_{\mathrm{CIE}}\left(j^{\prime }\right)$, in the course of the year 2018, see Figure 5b, but with the focus on a temporal and not on a SZA dependence. All ratio curves are virtually flat, and they stabilize by 1 July at the latest to their annual mean values as given in Table 3. The Genicom detecting system has the highest percentage standard deviation with 7% followed by PSF with 5%. As expected from their daily $r_{X\left(\lambda \right),\mathrm{CIE}}$ courses, the VioSpor dosimeter’s and the JEC1-IDE radiometer’s 2018 ratios are close to 1. Overall, the annual standard deviations demonstrate that the mean ratios can be applied as correction factors the better the longer the devices are measuring.

None of these detectors has been intended to mimic relative spectral sensitivities other than the CIE standard erythema curve, but additional ratios can be calculated regarding the NMSC, ICNIRP, and Setlow weighting function to provide insights in their comparability with each other. The mean annual ratio values $r_{X\left(\lambda \right),\mathrm{NMSC}}$, $r_{X\left(\lambda \right),\mathrm{ICNIRP}}$, and $r_{X\left(\lambda \right),\mathrm{Setlow}}$ are presented in Table 3. Although irradiances measured by the 501-UV biometer are disproportionately high by a factor of approximately 2 regarding $E_{\mathrm{CIE}}$, they are appropriate to describe $E_{\mathrm{NMSC}}$ without any correction. In contrast, the VioSpor and JEC1-IDE detectors underestimate $E_{\mathrm{NMSC}}$. A constant factor of ~0.5 is present for all $r_{X\left(\lambda \right),\mathrm{NMSC}}$ when compared to $r_{X\left(\lambda \right),\mathrm{CIE}}$ because of their annual UV doses, which are given by 1.0 MJm^−2^ (NMSC) and 484 kJm^−2^ (CIE). For UV doses weighted with the ICNIRP and Setlow action spectrum, these factors are 4.3 and 0.02, respectively.

Overall, the annual ratios are mean values taking all SZAs into account. Consequently, where the ratio strongly varies with the position of the sun, as we have mentioned above for the Setlow action spectrum, the averaged values do not adequately represent each daily situation, for example, at noon (minimum SZA) or in the early morning/late afternoon (maximum SZA). This mean nature is also apparent from the relatively high standard deviations in the case of the Setlow action spectrum (19–25% instead of 2–12% for the other weighting functions).

We conclude that the ratios of UV irradiances (or doses) from several detectors and erythemally weighted ones are practically constant throughout the day and the year. The detectors can therefore provide accurate risk estimates for erythema and NMSC when corrected with constant factors supporting the comparability of differently recorded data in the literature. They are not suitable to predict melanoma risk (if Setlow’s action spectrum is correct).

## 4. Discussion

The irradiances and related doses weighted to account for different biological effects or detected by several devices vary considerably depending on how the action spectra or detector responsivities overlap with the solar spectra. Between NMSC induction and erythema, for instance, the discrepancy is by a factor of about 2 regarding their cumulative annual UV doses (1.0 MJm^−2^ compared to 484 kJm^−2^). That is reasonable as the carcinogenesis action spectrum is shifted towards higher wavelengths by a few nanometers. Regarding the detectors, the JEC1-IDE system and the VioSpor dosimeter mimic the erythema response quite reliably, whereas the UV Biometer 501 measures UV irradiances (and UV doses) closer to the NMSC weighted values. That again makes sense because the shift of the UV Biometer 501 responsivity against the standard erythema spectrum is similar to that against the carcinogenesis action spectrum. Considerably higher weighted irradiances are found with the RBM, the PSF, and the Genicom detectors. For these three detecting systems this is due to the pronounced “shoulder” of their responsivity curves, which give undue weight to the spectral region between approximately 300 and 315 nm where the other spectra show a (close to) exponential decline.

If the ratio between detected irradiances and carcinogenesis or erythema weighted ones was the same for all kinds of solar spectra, one could simply apply a constant factor to correct RBM or PSF measurements in order to obtain values representative of the biologically weighted irradiances. Unfortunately, this is not the case. The ratio, for instance, of RBM to CIE erythema weighted irradiances varies from 6 to 8 between 30° and 90° SZA at 51.5° northern latitude (Dortmund, Germany), or from 10 to 13 between 20° and 70° SZA at 19.3° southern latitude (Townsville, Australia). For PSF detectors the ratio ranges from 11 to 18 at latitude 51.5° N and from 13 to 20 at latitude 20° S. Similar uncertainties of the correction factors for PSF exposures were calculated by Knuschke and Barth, 1996 [48]. The authors argued that PSF is easy to handle and inexpensive, and so it would still be appropriate for routine personal UVR dosimetry in spite of the described inaccuracies. Many other scientists have resorted to PSF dosimetry when they wanted to assess individual exposures, especially on different parts of the body [52,53,54,55].

With small size electronic or spore film detectors having become available more and more over the last two decades, we are no longer dependent on devices for which calibration factors can vary. The JEC1-IDE sensor, the UV Biometer 501, and the VioSpor dosimeter give readings, which are very similar to erythema or carcinogenesis weighted irradiances calculated from spectral measurements by convolution. Even the Genicom detector seems adequate, although its readings are a factor of 5 higher than the spectrally determined erythema weighted ones, but at least it seems possible to apply a single calibration factor for the most relevant SZAs between 20° and 70°.

Generally, it can be stated that the larger the spectral responsivity is in the UV-A range, the less congruent are the results for weighting, or accumulating measurements with the results of erythema weighting by spectral convolution. This is the reason for the Genicom detector being able to give adequate results: although its responsivity does not mimic the standard CIE erythema sensitivity very closely, it has the advantage of “ignoring” UVR above 320 nm. The RBM and the PSF suffer precisely from this problem of being overly sensitive in the UV-A range.

In the introduction, we mentioned that one purpose of this study was to see if variations of skin cancer with irradiances either calculated assuming biological weighting functions or measured with certain UVR dosimeters could be used to extract a general dose-effect relationship between UVR exposure and skin cancer risk. Such data are available, for instance, from a number of studies of skin cancer incidence in dependence of latitude. Of course, wherever data on ambient UV are used, there is still the open question of their relationship with individual doses, but we are at least one step closer to a realistic dose-effect relationship. Some studies have already focused on individual UVR exposure, and with the increased availability of electronic UVR dosimeters this approach will play an even greater role in the future.

Our results show that the most decisive parameter that weighted exposures depend on is the SZA, whereas season or latitude are of minor importance. We did not consider the influence of other factors, such as stratospheric ozone level [56,57], cloudiness [56,57,58], air pollution [56,59], ground albedo [60], or altitude above sea level [61]. All of these factors can considerably modify the solar spectrum, but whether the associated variations in erythemally weighted exposures parallel those measured with UVR dosimeters in a similar way as we have shown for the SZA is a matter to be further investigated.

The conclusion that the SZA is of decisive importance holds true irrespective of weighted exposures being calculated either with CIE’s standard curves for erythema or for NMSC or measured with one of the more adequate devices (as just discussed). In contrast, RBM and even more so PSF dosimeters have a somewhat different SZA dependence than, for example, CIE weighted irradiances, as can be seen in the daily courses of $r_{\mathrm{RBM},\mathrm{CIE}}$ and $r_{\mathrm{PSF},\mathrm{CIE}},\;$ which suggests that these still popular devices should be employed with care because their use may lead to somewhat distorted results.

Serious doubts must be raised whether calculations based on the erythema standard curve or similar action spectra, or measurements with detectors designed to mimic the erythema response, can help to establish a dose–effect relationship for UVR induced melanoma. Whereas the erythemally weighted irradiances vary by a factor of 12 between 2° and 70° SZA the melanoma effective irradiances only change by a factor of 4. Of course, this depends on the suitability of the action spectrum proposed by Setlow, 1999 [27]. It has been criticized because it is based on melanoma induction in fish, which may involve different mechanisms than in humans [62], and because of the doubtable statistical power of the experimental evidence [63,64]. Moreover, a study with transgenic mice suggested that melanoma induction by UV-A with wavelengths longer than 320 nm is negligible, which would speak in favor of an action spectrum similar to that of NMSC [65]. More recently, however, the same group has published results from experiments with a different strain of transgenic mice, arguably more closely modeling human skin with respect to melanogenesis. The evidence presented supports Setlow’s suggestion that melanoma induction by UV-A does require the presence of melanin [66]. Therefore, his action spectrum, although being very different from all others, should not be dismissed without careful consideration. If it does apply, then all our devices that are carefully designed to reflect erythema (or NMSC) sensitivity as closely as possible, cannot be expected to give reliable information about melanoma risks.

## 5. Conclusions

UV irradiances and doses measured with any of the detectors used in this study are able to provide quite accurate estimates of solar UVR effective for erythema and NMSC induction or for ICNIRP’s relative spectral UVR sensitivity, as long as certain correction factors are applied. With the more “traditional” devices, namely the Robertson–Berger meter and the polysulphone film, these correction factors vary by roughly 20% between SZAs of 60° and 30° (calculations based on erythemal sensitivity). For more recently developed devices, however, the correction factors are fairly constant throughout the day and year. They are close to 1 for the JEC1-IDE and the VioSpor UVR dosimeters. All this may not apply for melanoma induction, if, as a number of studies suggest, it is substantially influenced by melanin absorption. We conclude that it is possible to meaningfully compare data from studies correlating UV doses and (non-melanoma) skin cancer even if different dosimeters have been used, as long as these have a sufficiently similar spectral response.

## Figures and Tables

**Table 1:** Information on the action spectra and detector responsivities presented in Figure 1 and Figure 2, respectively.

	Description
CIE	standard erythema curve according to Equation (3) established by CIE [24]
NMSC	CIE standard curve for non-melanoma skin cancer [25]
SCUP-h	experimental data for NMSC induction in mice corrected for differences in mouse and human epidermal transmission, taken from Table 1 in [23] and interpolated semi-logarithmically in steps of 1 nm
ICNIRP	“UVR hazards curve” from ICNIRP [31] according to Equation (4)
Setlow	combined absorption spectrum of DNA and melanin multiplied with human epidermal transmission as given in Figure 1 of [27], digitized in 1 nm intervals
RBM	Robertson–Berger meter response function taken from Table 2 of [41], interpolated semi-logarithmically in steps of 1 nm
501-UV	Solar Light’s UV Biometer 501, representing a modified RBM, taken from the manufacturer’s webpage [42], digitized in 1 nm intervals
JEC1-IDE	UVR radiometer based on a silicon carbide photodiode with dielectric filter taken from Figure 2 of [43], digitized and interpolated semi-logarithmically in steps of 1 nm
Genicom	UVR radiometer based on an aluminum gallium nitride photodiode from Figure S1 of [44], digitized and interpolated semi-logarithmically in steps of 1 nm
PSF	CIE standard polysulfone film dosimeter from Figure 4.4 of [45], digitized and interpolated semi-logarithmically in steps of 1 nm
VioSpor	spore film dosimeter, mutant strain modulated with a cut-off filter, taken from Figure 3 of [46], digitized and interpolated semi-logarithmically in steps of 1 nm

**Table 2:** Factors for a renormalization of all action spectra to the value at 297 nm.

	Peak Sensitivity at *λ* (nm)	Renormalization Factor
CIE	≤298	1.00
NMSC	299	0.96
ICNIRP	270	0.41
Setlow	293	0.75
RBM	300	0.95
501-UV	292	0.89
JEC1-IDE	298.5	1.19
Genicom	≤296	0.99
PSF	290	0.99
VioSpor	300	1.05

**Table 3:** Mean values with standard deviations of the ratios of measured to weighted UV doses for 2018. The $r_{X\left(\lambda \right),\mathrm{CIE}}$ values in column two reflect the data given in Figure 5b.

	CIE	NMSC	ICNIRP	Setlow
PSF	16.2 ± 0.8	8 ± 1	70 ± 5	0.37 ± 0.07
RBM	7.9 ± 0.1	4.0 ± 0.3	34 ± 1	0.18 ± 0.04
Genicom	5.4 ± 0.4	2.69 ± 0.03	23 ± 1	0.12 ± 0.03
501-UV	1.93 ± 0.06	0.97 ± 0.05	8.4 ± 0.2	0.04 ± 0.01
VioSpor	1.09 ± 0.02	0.55 ± 0.03	4.73 ± 0.03	0.025 ± 0.006
JEC1-IDE	0.95 ± 0.03	0.48 ± 0.02	4.12 ± 0.09	0.022 ± 0.005

## References

[1] De Gruijl F.R. (1999). Skin cancer and solar UV radiation. Eur. J. Cancer.

[2] Reeve V.E., Ley R.D., Hill D., Elwood J.M., English D.R. (2004). Animal models of ultraviolet radiation-induced skin cancer. Prevention of Skin Cancer—Cancer Causes.

[3] National Research Council (NRC) (1984). Causes and Effects of Changes in Stratospheric Ozone: Update 1983.

[4] Qureshi A.A., Laden F., Colditz G.A., Hunter D.J. (2008). Geographic variation and risk of skin cancer in US women. Differences between melanoma, squamous cell carcinoma, and basal cell carcinoma. Arch. Intern. Med..

[5] Moan J., Grigalavicius M., Baturaite Z., Juzeniene A., Dahlback A. (2013). North-South gradients of melanomas and non-melanomas: A role of vitamin D?. Dermatoendocrinology.

[6] Rivas M., Rojas E., Calaf G.M., Barberán M., Liberman C., De Paula Correa M. (2017). Association between non-melanoma and melanoma skin cancer rates, vitamin D and latitude. Oncol. Lett..

[7] Bauer A., Diepgen T.L., Schmitt J. (2011). Is occupational solar ultraviolet irradiation a relevant risk factor for basal cell carcinoma? A systematic review and meta-analysis of the epidemiological literature. Br. J. Dermatol..

[8] Modenese A., Korpinen L., Gobba F. (2018). Solar Radiation Exposure and Outdoor Work: An Underestimated Occupational Risk. Int. J. Environ. Res. Public Health.

[9] Paulo M.S., Adam B., Akagwu C., Akparibo I., Al-Rifai R.H., Bazrafshan S., Gobba F., Green A.C., Ivanov I., Kezic S. (2019). WHO/ILO work-related burden of disease and injury: Protocol for systematic reviews of occupational exposure to solar ultraviolet radiation and of the effect of occupational exposure to solar ultraviolet radiation on melanoma and non-melanoma skin cancer. Environ. Int..

[10] Moehrle M. (2008). Outdoor sports and skin cancer. Clin. Dermatol..

[11] Li J., Uter W., Pfahlberg A., Gefeller O. (2012). A comparison of patterns of sun protection during beach holidays and everyday outdoor activities in a population sample of young German children. Br. J. Dermatol..

[12] Diffey B.L. (2018). Time and Place as Modifiers of Personal UV Exposure. Int. J. Environ. Res. Public Health..

[13] Gandini S., Sera F., Cattaruzza M.S., Pasquini P., Picconi O., Boyle P., Melchi C.F. (2005). Meta-analysis of risk factors for cutaneous melanoma: II. Sun exposure. Eur. J. Cancer..

[14] Xiang F., Lucas R., Hales S., Neale R. (2014). Incidence of nonmelanoma skin cancer in relation to ambient UV radiation in white populations, 1978–2012: Empirical relationships. JAMA Dermatol..

[15] Wu S., Han J., Vleugels R.A., Puett R., Laden F., Hunter D.J., Qureshi A.A. (2014). Cumulative ultraviolet radiation flux in adulthood and risk of incident skin cancers in women. Br. J. Cancer..

[16] Zhu G.A., Raber I., Sakshuwong S., Li S., Li A.S., Tan C., Chang A.L. (2016). Estimation of individual cumulative ultraviolet exposure using a geographically-adjusted, openly-accessible tool. BMC Dermatol..

[17] Liu-Smith F., Farhat A.M., Arce A., Ziogas A., Taylor T., Wang Z., Yourk V., Liu J., Wu J., McEligot A.J. (2017). Gender differences in the association of cutaneous melanoma incidence rates and geographic ultraviolet light exposure. J. Am. Acad. Dermatol..

[18] Vernez D., Milon A., Vuilleumier L., Bulliard J.L. (2012). Anatomical exposure patterns of skin to sunlight: Relative contributions of direct, diffuse and reflected ultraviolet radiation. Br. J. Dermatol..

[19] Weihs P., Schmalwieser A.W., Reinisch C., Meraner E., Walisch S., Harald M. (2013). Measurements of personal UV exposure on different parts of the body during various activities. Photochem. Photobiol..

[20] Casale G.R., Siani A.M., Diémoz H., Kimlin M.G., Colosimo A. (2012). Applicability of the polysulphone horizontal calibration to differently inclined dosimeters. Photochem. Photobiol..

[21] Schmalwieser A.W., Casale G.R., Colosimo A., Schmalwieser S.S., Siani A.M. (2021). Review on Occupational Personal Solar UV Exposure Measurements. Atmosphere.

[22] Schmalwieser A.W., Siani A.M. (2018). Review on Nonoccupational Personal Solar UV Exposure Measurements. Photochem. Photobiol..

[23] de Gruijl F.R., Van der Leun J.C. (1994). Estimate of the Wavelength Dependency of Ultraviolet Carcinogenesis in Humans and Its Relevance to the Risk Assessment of a Stratospheric Ozone Depletion. Health Phys..

[24] International Organization for Standardization/International Commission on Illumination (2019). ISO/CIE 17166:2019 Erythema Reference Action Spectrum and Standard Erythema Dose. https://www.iso.org/standard/74167.html.

[25] International Organization for Standardization/International Commission on Illumination (2016). ISO/CIE 28077:2016 Photocarcinogenesis Action Spectrum. https://www.iso.org/standard/69651.html.

[26] Parisi A.V., Wong J.C. (1997). Erythemal irradiances of filtered ultraviolet radiation. Phys. Med. Biol..

[27] Setlow R.B. (1999). Spectral Regions Contributing to Melanoma: A Personal View. J. Investig. Derm. Symp. Proc..

[28] Madronich S., Young R.A., Björn L.O., Moan J., Nultsch W. (1993). The Atmosphere and UV-B Radiation at Ground Level. Environmental UV Photobiology.

[29] Bernhard G., Mayer B., Seckmeyer G., Moise A. (1997). Measurements of spectral solar UV irradiance in tropical Australia. J. Geophys. Res..

[30] Gerstl S.A.W., Zardecki A., Wiser H.L. (1983). UV-B Handbook, Vol. I.

[31] International Commission on Non-Ionizing Radiation Protection (ICNIRP) (2004). Guidelines on Limits of Exposure to Ultraviolet Radiation of Wavelengths between 180 nm and 400 nm (Incoherent Optical Radiation). Health Phys..

[32] McKinlay A.F., Diffey B.L. (1987). A reference action spectrum for ultraviolet induced erythemal in human skin. CIE J..

[33] Webb A.R., Slaper H., Koepke P., Schmalwieser A.W. (2011). Know your standard: Clarifying the CIE erythema action spectrum. Photochem. Photobiol..

[34] Schmalwieser A.W., Wallisch S., Diffey B. (2012). A Library of Action Spectra for Erythema and Pigmentation. Photochem. Photobiol. Sci..

[35] International Commission on Illumination (2003). CIE S 013/E:2003 International Standard Global Solar UV Index. https://cie.co.at/publications/international-standard-global-solar-uv-index.

[36] Setlow R.B., Grist E., Thompson K., Woodhead A.D. (1993). Wavelengths effective in induction of malignant melanoma. Proc. Natl. Acad. Sci. USA.

[37] Anderson R.R., Parrish J.A. (1981). The Optics of Human Skin. J. Investig. Dermat..

[38] Bruls W.A., Slaper H., van der Leun J.C., Berrens L. (1984). Transmission of human epidermis and stratum corneum as a function of thickness in the ultraviolet and visible wavelengths. Photochem. Photobiol..

[39] Meinhardt M., Krebs R., Anders A., Heinrich U., Tronnier H. (2008). Effect of ultraviolet adaptation on the ultraviolet absorption spectra of human skin in vivo. Photodermatol. Photoimmunol. Photomed..

[40] Berger D.S. (1976). The sunburning ultraviolet meter: Design and performance. Photochem. Photobiol..

[41] De Luisi J., Wendell J., Kreiner F. (1992). An Examination of the Spectral Response Characteristics of Seven Robertson-Berger Meters After Long-Term Field Use. Photochem. Photobiol..

[42] Solar Light User Manual Model 501-DA Analog UV Biometer Sensor. https://solarlight.com/wp-content/uploads/Model-501-DA-Analog-UV-Biometer-User.pdf.

[43] Heydenreich J., Wulf H.C. (2019). Personal UVR Dosimeter Measurements: Specific and General Uncertainties. Photochem. Photobiol. Sci..

[44] Grandahl K., Erikson P., Ibler K.S., Bonde J.P., Mortensen O.S. (2018). Supporting Information Measurements of Solar Ultraviolet Radiation Exposure at Work and at Leisure in Danish Workers. Photochem. Photobiol..

[45] International Commission on Illumination (CIE) (1992). Technical Report—Personal Dosimetry of UV Radiation, CIE 98. https://cie.co.at/publications/personal-dosimetry-uv-radiation.

[46] Quintern L.E., Furusawa Y., Fukutsu K., Holtschmidt H. (1997). Characterization and Application of UV Detector Spore Films: The Sensitivity Curve of a New Detector System Provides Good Similarity to the Action Spectrum for UV-Induced Erythema in Human Skin. J. Photochem. Photobiol. B.

[47] Morys M., Berger D.S., Stamnes K.H. (1993). The accurate measurements of biologically effective ultraviolet radiation. Atmospheric Radiation.

[48] Grandahl K., Mortensen O.S., Sherman D.Z., Køster B., Lund P.A., Ibler K.S., Eriksen P. (2017). Solar UV exposure among outdoor workers in Denmark measured with personal UV-B dosimeters: Technical and practical feasibility. Biomed. Eng. Online.

[49] Davis A., Deane G.H., Diffey B.L. (1976). Possible dosimeter for ultraviolet radiation. Nature.

[50] Knuschke P., Barth J. (1996). Biologically weighted personal UV dosimetry. J. Photochem. Photobiol. B.

[51] Casale G.R., Siani A.M., Colosimo A. (2009). Polysulphone dosimetry as a tool for personal exposure studies. Biophys. Bioeng. Lett..

[52] Kimlin M.G., Martinez N., Green A.C., Whiteman D.C. (2006). Anatomical distribution of solar ultraviolet exposures among cyclists. J. Photochem. Photobiol. B.

[53] Downs N., Parisi A. (2012). Mean exposure fractions of human body solar UV exposure patterns for application in different ambient climates. Photochem. Photobiol..

[54] Seckmeyer G., Klingebiel M., Riechelmann S., Lohse I., McKenzie R.L., Liley J.B., Allen M.W., Siani A.M., Casale G.R. (2012). A critical assessment of two types of personal UV dosimeters. Photochem. Photobiol..

[55] Siani A.M., Casale G.R., Modesti S., Parisi A.V., Colosimo A. (2014). Investigation on the capability of polysulphone for measuring biologically effective solar UV exposures. Photochem. Photobiol. Sci..

[56] Bais A.F., McKenzie R.L., Bernhard G., Aucamp P.J., Ilyas M., Madronich S., Tourpali K. (2015). Ozone depletion and climate change: Impacts on UV radiation. Photochem. Photobiol. Sci..

[57] Bais A.F., Bernhard G., McKenzie R.L., Aucamp P.J., Young P.J., Ilyas M., Jöckel P., Deushi M. (2019). Ozone-climate interactions and effects on solar ultraviolet radiation. Photochem. Photobiol. Sci..

[58] Aun M., Eerme K., Ansko I., Veismann U., Lätt S. (2011). Modification of spectral ultraviolet doses by different types of overcast cloudiness and atmospheric aerosol. Photochem. Photobiol..

[59] McKenzie R.L., Weinreis C., Johnston P.V., Liley B., Shiona H., Kotkamp M., Smale D., Takegawa N., Kondo Y. (2008). Effects of urban pollution on UV spectral irradiances. Atmos. Chem. Phys..

[60] Turner J., Parisi A.V., Turnbull D.J. (2008). Reflected solar radiation from horizontal, vertical and inclined surfaces: Ultraviolet and visible spectral and broadband behaviour due to solar zenith angle, orientation and surface type. J. Photochem. Photobiol. B.

[61] Utrillas M.P., Marín M.J., Esteve A.R., Salazar G., Suarez H., Castillo J., Martínez-Lozano J.A. (2016). UVER and UV index at high altitude in Northwestern Argentina. J. Photochem. Photobiol. B.

[62] von Thaler A.K., Kamenisch Y., Berneburg M. (2010). The role of ultraviolet radiation in melanomagenesis. Exp. Dermatol..

[63] Mitchell D., Paniker L., Sanchez G., Trono D., Nairn R. (2007). The etiology of sunlight-induced melanoma in Xiphophorus hybrid fish. Mol. Carcinog..

[64] Mitchell D.L., Fernandez A.A., Nairn R.S., Garcia R., Paniker L., Trono D., Thames H.D., Gimenez-Conti I. (2010). Ultraviolet A does not induce melanomas in a Xiphophorus hybrid fish model. Proc. Natl. Acad. Sci. USA.

[65] De Fabo E.C., Noonan F.P., Fears T., Merlino G. (2004). Ultraviolet B but not ultraviolet A radiation initiates melanoma. Cancer Res..

[66] Noonan F.P., Zaidi M.R., Wolnicka-Glubisz A., Anver M.R., Bahn J., Wielgus A., Cadet J., Douki T., Mouret S., Tucker M.A. (2012). Melanoma induction by ultraviolet A but not ultraviolet B radiation requires melanin pigment. Nat. Commun..

